# Contingent negative variation during a modified cueing task in simulated driving

**DOI:** 10.1371/journal.pone.0224966

**Published:** 2019-11-11

**Authors:** Zizheng Guo, Xi Tan, Yufan Pan, Xian Liu, Guozhen Zhao, Lin Wang, Zhen Peng

**Affiliations:** 1 School of Transportation and Logistics, Southwest Jiaotong University, Chengdu, China; 2 National United Engineering Laboratory of Integrated and Intelligent Transportation, Southwest Jiaotong University, Chengdu, China; 3 National Engineering Laboratory for Comprehensive Transportation Big Date Application Technology, National Development and Reform Commission, Beijing, China; 4 CAS Key Laboratory of Behavioral Science, Institute of Psychology, Beijing, China; 5 School of Arts and Sciences, Arizona State University, Tempe, Arizona, United States of America; Tongii University, CHINA

## Abstract

The obscured pedestrian-motor vehicle crash has become a serious danger to driving safety. The present study aims to investigate the contingent negative variation (CNV) during the anticipation of an obscured pedestrian-motor vehicle crash in simulated driving. We adopted two cueing tasks: (i) a traditional cognitive paradigm of cueing task that has been widely used to study anticipatory process, and (ii) a modified cueing task in simulated driving scenes, in which Electroencephalogram (EEG) signals of 32 participants were recorded to detect the CNV. Simulated car following and pedestrian crossing tasks were designed to measure anticipation-related driving behaviors. The results showed that both early and late CNVs were observed in two cueing tasks. The mean amplitude of the late CNV during a modified cueing task in simulated driving was significantly larger than that in a traditional cueing task, which was not the case for the early CNV potentials. In addition, both early and late CNVs elicited in simulated driving were significantly correlated with anticipatory driving behaviors (e.g., the minimum time to collision). These findings show that CNV potentials during the anticipation of an obscured pedestrian-motor vehicle crash might predict anticipation-related risky driving behaviors.

## Introduction

The obscured pedestrian-motor vehicle crash refers to the scenario in which a pedestrian pops up from the blind spot generated by a parked vehicle in front of the driver. This type of crash has become a serious hidden danger to driving safety in recent years. According to the National Highway Traffic Safety Administration (NHTSA), pedestrians were obscured from drivers’ views in 13 percent of all pedestrian involvements and 17 percent of pedestrian deaths in the United States from 2005 to 2009 [1]. In China, more than 200,000 people die in traffic accidents every year due to obscured pedestrian-motor vehicle crashes [2], and obscured pedestrian-motor vehicle crashes usually occur at bus stations due to the large blind area created by buses [3]. According to one study of pedestrian safety, the rapid appearance of pedestrians and their short-term exposure to drivers are two key factors that cause obscured pedestrian-motor vehicle crashes [4]. For example, the stopped vehicle may block the view of the driver of an oncoming car who plans to pass the stopped vehicle, which puts a rushing pedestrian at risk of being hit.

Two categories of solutions have been proposed to prevent such obscured pedestrian-motor vehicle crashes. From an environmental perspective, blind spots are created by surrounding vehicles, and it is difficult for drivers to pay attention to hidden pedestrians when blind spots occur [5, 6]. Therefore, the in-vehicle head-up display (HUD) system [7] and the autonomous emergency braking (AEB) system [8] are designed to facilitate the detection of hidden vehicles and pedestrians and to apply emergency brakes if a driver does not respond. The previous experimental results showed that the implementation of diagonal parking significantly increased the number of pedestrians scanning for traffic before entering the roadway [9]. From a driver’s perspective, on the other hand, driving history and experience are associated with a driver’s anticipation of an obscured pedestrian-motor vehicle crash. Compared to novice drivers, experienced drivers are more likely to scan the left front edge of a truck for possible pedestrians when approaching the truck [10]. Experienced drivers are good at watching out for those areas of a scenario from which hidden risks could emerge. For example, a parked bus at a bus station can serve as a reminder for experienced drivers to anticipate possible obscured pedestrians and to prepare to brake in advance [11, 12]. Using a PC-based program to train novice drivers to recognize hidden risks early on significantly improved their awareness of hazards, both on an advanced driving simulator and on the road [13–15].

The present study attempts to investigate the neural mechanism underlying the anticipation of an obscured pedestrian-motor vehicle crash. In the area of cognitive psychology, anticipation refers to the situation in which people await the presentation of a stimulus and focus on the input side of information processing [16, 17], such as the perception of a pedestrian popping up from a blind spot. The cueing paradigm is a classical experimental paradigm for studying the cognitive process of anticipation and has been widely studied [18–21]. In the field of classic experimental psychology, the cognitive process of anticipation has been widely studied in a cueing paradigm [17]. This paradigm consists of paired stimuli: the first stimulus is a cue that activates an individual’s anticipation and action preparation; the second stimulus is a target that requires him/her to make a response [22]. For example, when the cue stimulus is presented, the participant is required to prepare for the keystroke. After an inter-stimuli interval (e.g., ISI = 3 s), the target stimulus is presented, and the participant is required to press the key as soon as possible [23].

During the study of anticipation, contingent negative variation (CNV), a negative event-related potential (ERP) component, was first detected between the cue stimuli and target stimuli in [17]. A single flash or electrical stimulus was presented as cue stimuli, then the CNV was presented approximately 1s after the cue stimuli onset. EEG signals were recorded at several electrode sites and the maximum amplitude of CNV was observed in the central and frontal cortex (e.g., Cz) [17, 24]. The CNV waveform reflects the cognitive processes of attention, anticipation, and preparation to target stimuli. In addition to the classic cueing paradigm, CNV was also detected by combing high-density electroencephalogram (EEG) recordings with the variant of the above cuing paradigm. A relatively long inter-stimulus interval (i.e., 3–4 s between a cue and a target stimulus) revealed that the CNV consists of at least two components, the early and late CNVs over the central and frontal cortex [25–28]. The early CNV was sensitive to the intensity and sensory modality of cue stimuli. For example, in the pairs of pure tones stimuli, 12 participants were aroused the alerting properties when the presentation of high-frequency cue stimuli. The early CNV, maximal over the frontal parietal areas (e.g., Fz), was observed and persisted for approximately 1.2–1.5 s [18,19]. On the other hand, the late CNV began to develop about 1s prior to target stimuli and peaked at about the time of target stimuli appearing. The late CNV was associated with anticipatory attention to the target stimuli and the preparation of the motor response, which was maximal over the central parietal areas (e.g., Cz and Pz) [19, 20, 29–31]. Although none of the studies above are aimed at drivers, the existence of CNVs provides a new possibility for detecting a driver’s unawareness of an obscured pedestrian-motor vehicle crash.

Khaliliardali, Chavarriaga [32] have investigated anticipation as the cognitive state leading to specific actions during simulated driving. The authors combined a variant of the CNV paradigm with the task of inhibitory control to recognize a driver’s intention through anticipatory brain potentials. In their experiment, when participants were driving a virtual car along a highway, a countdown (cue stimulus) appeared at the center of the screen from ‘4’ to ‘1’, indicating that a Go/Stop cue (target stimulus) would appear after driving 4000 ms. The appearance of the Go cue required the participants to push the gas pedal promptly, and the appearance of the Stop cue required the participants to push the brake pedal promptly. This modified cueing paradigm in a simulated driving task successfully elicited the CNV at Cz electrode around 2500 ms later after the cue stimuli onset. Later, the authors used the same experimental settings to predict actions during simulated driving [33]. Through a quadratic discriminant analysis classifier based on temporal features, decoding performance was reported before the action onset. The results showed that the centro-medial anticipatory potentials were observed as early as 320 ± 200 ms before the action with a detection rate of 0.77 ± 0.12 in the offline analysis.

The studies mentioned above contributed to achieving better communication between the driver and the intelligent car by recognizing a driver’s awareness of the anticipatory brain potential and laid a research basis for detecting CNV in the present study. However, the previous cueing paradigm in simulated driving (e.g., count down from 4 to 1 in [32, 33] or car crash end trial in [34]) was not a common scenario in actual driving, and the elicited CNV may be different from that during anticipation of an obscured pedestrian-motor vehicle with higher ecological validity. Moreover, previous studies did not examine whether the elicited CNV was able to predict anticipation-related driving behaviors.

In view of this, the current study used a parked bus at a bus station as the cue stimulus to create a blind spot, designed a modified cueing paradigm in simulated driving (a pedestrian suddenly crosses the road in front of the bus) to elicit the CNV, and applied two simulated driving tasks (car following and pedestrian crossing) to measure anticipation-related driving behaviors. The purposes of the current study were to investigate (i) whether the CNV can be observed in the modified cueing paradigm in simulated driving, (ii) whether the CNV induced by the anticipation of an obscured pedestrian-motor vehicle was different from that induced by the traditional cognitive paradigm of a cueing task, and (iii) whether the CNV during anticipation of an obscured pedestrian-motor vehicle was associated with other anticipation-related risky driving behaviors.

## Materials and methods

### Participants

Thirty-four volunteers responded to the recruitment advertisements. We excluded two participants due to insufficient EEG artifact-free trials (less than 5 trials) or EEG data recording problems. As a result, thirty-two of the volunteers (28 males and 4 females, average age = 23.41, SD = 1.93 years) were selected to participate in this study. All of the participants were right-handed and had normal or corrected-to-normal vision. They had valid Chinese driver licenses with at least one year of licensed driving experience and had driven at least twice per week in the past month. Their average driving mileage was 2996.88 (SD = 3732.33) km. In addition, all participants had no history of psychiatric disorders or substance addiction. This study was approved by the Ethics Committee of Human Experimentation at the Institute of Psychology, Chinese Academy of Sciences. All research activities were performed according to relevant guidelines and regulations outlined in the approved protocol.

### Experimental tasks

All participants completed two cueing tasks and two simulated driving tasks. The two cuing tasks consisted of one traditional cognitive paradigm of the cueing task and one modified paradigm of the cueing task in simulated driving scenes. Two simulated driving tasks included a car-following (CF) driving scenario and a pedestrian-crossing (PC) driving scenario.

#### Cognitive paradigm of the cueing task

**Fig 1:**
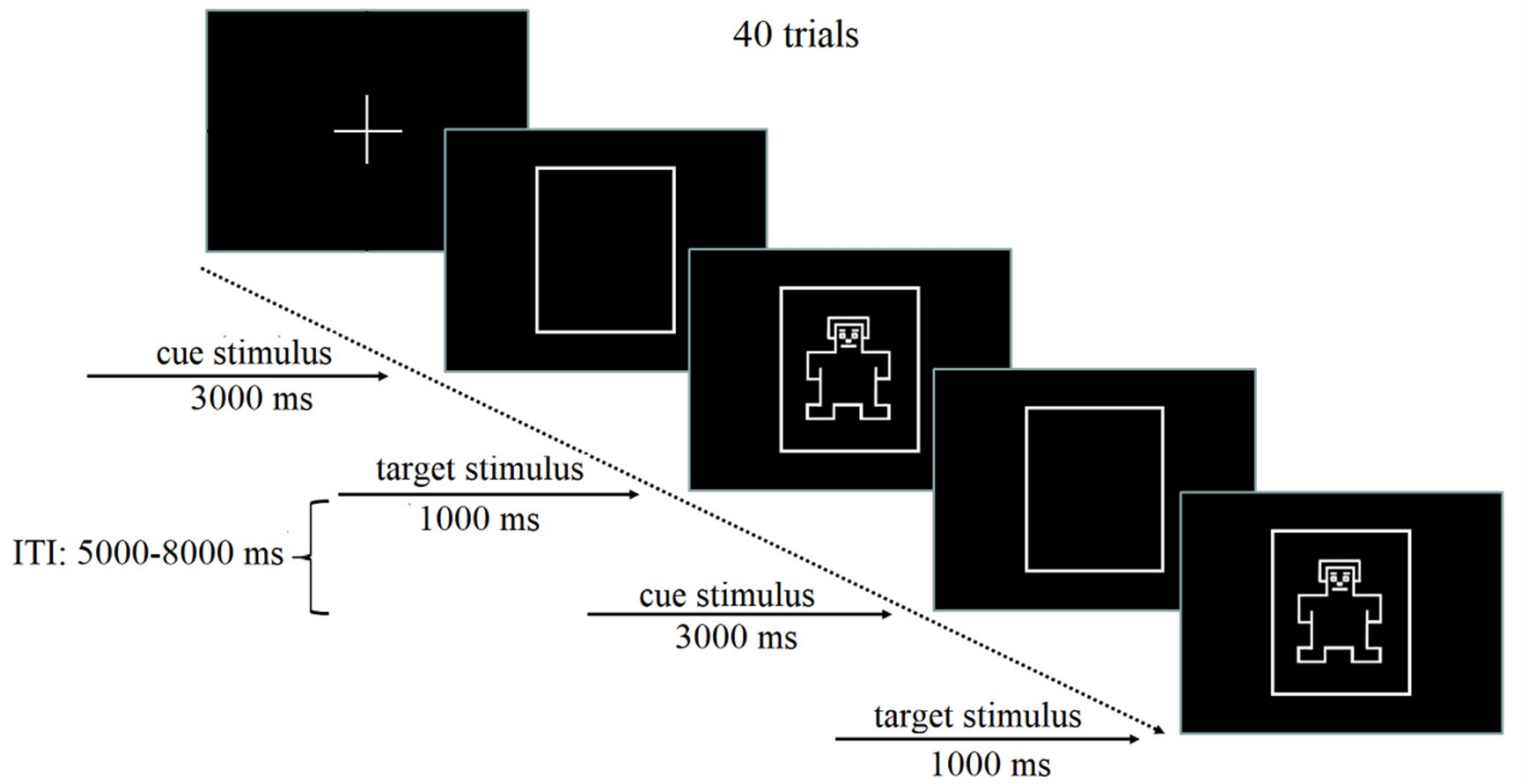
Schematic diagram showing the timing of the cognitive paradigm of a cueing task. A square symbol was presented in the center for 3000 ms as the cue stimulus. A small person symbol (target stimulus) appeared in the center of the square. The participants were asked to press the corresponding button as soon as the target stimulus was presented.

The cognitive paradigm of the cueing task in the baseline condition has been widely used to study the cognitive process of anticipation. In this paradigm, the cue stimulus is first presented to activate an individual’s anticipation and action preparation. After an ISI, the target stimulus is presented to require him/her to make a response. As illustrated in Fig 1, each trial started with a centrally presented square cue stimulus (10×10 cm, 3000-ms duration, with an approximate visual angle of 6.68×6.68°), followed by a target stimulus (a symbolized person image, 1000-ms duration, with an approximate visual angle of 5×5°) that was presented in the center of the square. The interval between a cue stimulus and a target stimulus was 3 seconds. The participants were instructed to press the M key as quickly as possible without sacrificing accuracy when they saw the target stimuli. The next trial was followed by an inter-trial interval of 5~8 s. The baseline condition consisted of 40 trials.

#### Modified cueing task in simulated driving

**Fig 2:**
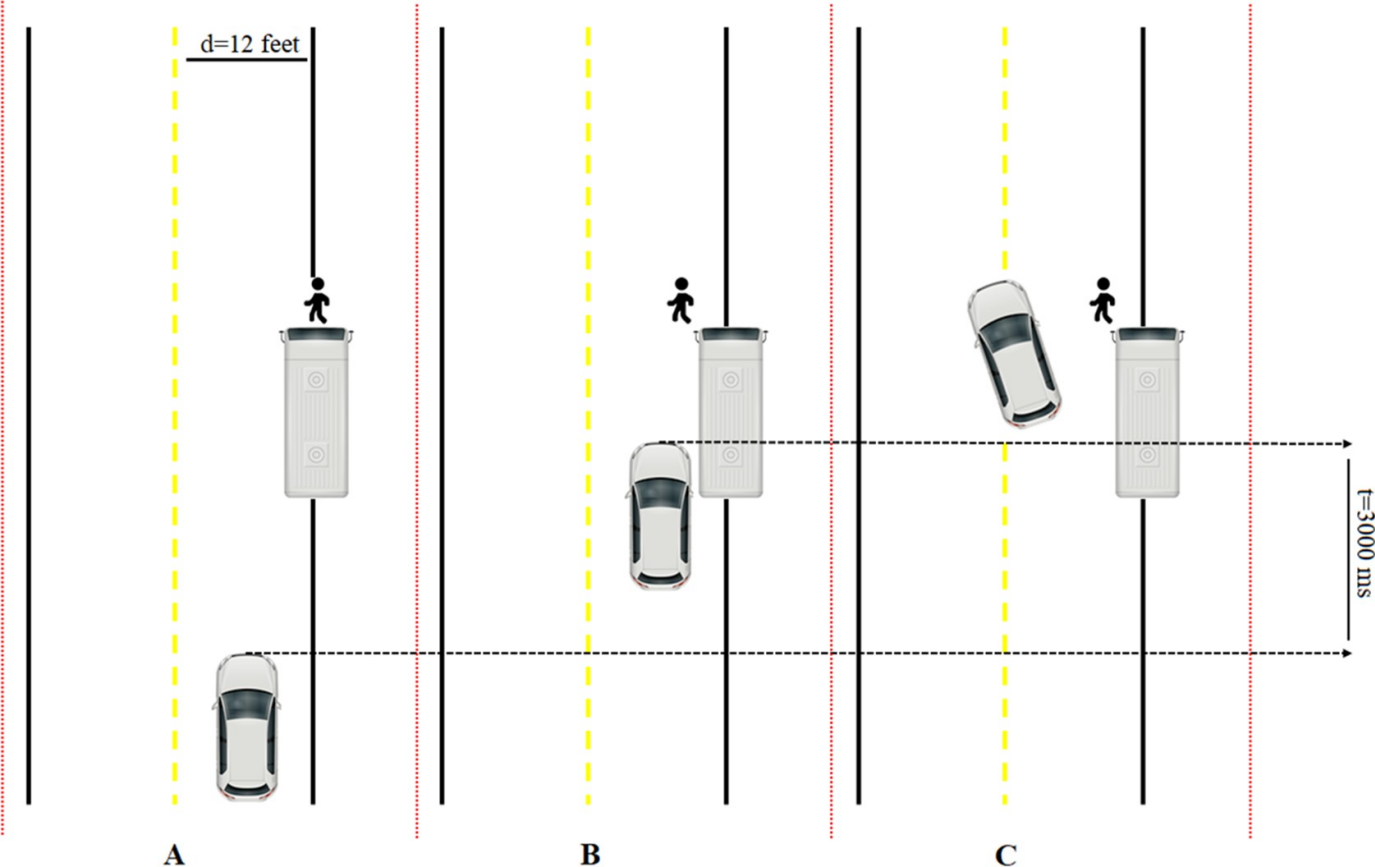
Schematic diagram of the timing of the modified cueing task in simulated driving. (A) The participants were instructed to drive in the middle of the right lane at a fixed speed of 25 mph. A parked bus appeared at the bus station as a cue stimulus, and a pedestrian was obscured by the bus. (B) After driving the simulator for 3 s, the pedestrian (target stimulus) would suddenly appear 1 foot from the left edge of the bus. (C) The participants were asked to turn the steering wheel towards the left to avoid hitting the simulated pedestrian and then to get the car back in the right lane as quickly as possible.

A modified cueing task was designed in the simulated driving environment. In this driving condition, participants were asked to operate the simulator on an urban two-lane road at a fixed speed of 25 mph. As shown in Fig 2, when the participants were driving in the middle of the right lane, a parked bus at a bus station (a cue stimulus by seeing of which the participants would anticipate a upcoming pedestrian) appeared, which indicated that an obscured pedestrian (a target stimulus by seeing of which the participants would react immediately) would suddenly appear in front of the parked bus (1 foot from the left edge of the bus, 65 feet from the moving car) after driving for 3000 ms. The target stimulus was presented after each cue stimulus, and the interval between the cue stimulus and target stimulus was 3 seconds. The appearance of the pedestrian required participants to slow down and/or steer the wheel quickly to avoid hitting the pedestrian. A single, broken yellow centerline (see Fig 2) permits passing on either side if safe conditions exist (i.e., there is no approaching vehicles in the other lane). If the collision occurred, there was a simulated collision frame with a broken front windshield and loud noises. The driving condition consisted of 40 trials. Each trial ended with participants getting the car back to the right lane, and then a new trial would start after 5~8 s.

#### Anticipation-related driving tasks

To investigate whether the CNV amplitude of the modified cueing task in driving condition can be used to predict more anticipation-related driving behaviors, two simulated driving tasks were designed: a car following task (CF task) and pedestrian crossing task (PC task).

***CF task*:** The participants were asked to operate the simulator to follow a leading vehicle with a fixed distance of 120 feet and to keep a constant speed of 45 mph. During driving, two road events could occur: (i) the leading vehicle sped up until it was out of view, or (ii) the leading vehicle suddenly braked until it stopped completely. At this point (indicated by the brake lights), the participants were required to brake as quickly as possible to avoid a collision. After a full stop, the leading vehicle accelerated away from the scene, and the participants were asked to increase their speed back to 45 mph. The leading vehicle was presented 24 times (the ITI was 1200 feet), and only half of them were braking trials (i.e., immediate brakes were needed) [35].

***PC task*:** The participants drove a car on the roadway with a limited speed of 25 mph. A simulated pedestrian would appear 2 feet from either the left or right roadside when the driver was 130 feet from the pedestrian. The pedestrian might start to cross the road with a constant speed (7 feet/s of left side, 5 feet/s of right side) when the driver was 130 feet from pedestrians on the left side and 70 feet from pedestrians on the right side. The side from which the pedestrian appeared and whether the pedestrian crossed the street were random. Therefore, the crossing pedestrians could be divided into 4 groups: (a) stationary pedestrian on the left side, (b) stationary pedestrian on the right side, (c) crossing pedestrian on the left side, and (d) crossing pedestrian on the right side. There were 30 simulated pedestrians in the PC task in 10 crossing conditions [36]. The participants were asked to avoid colliding with simulated pedestrians as if they were driving a real vehicle on the road and follow the traffic laws.

### Experimental procedures

**Fig 3:**
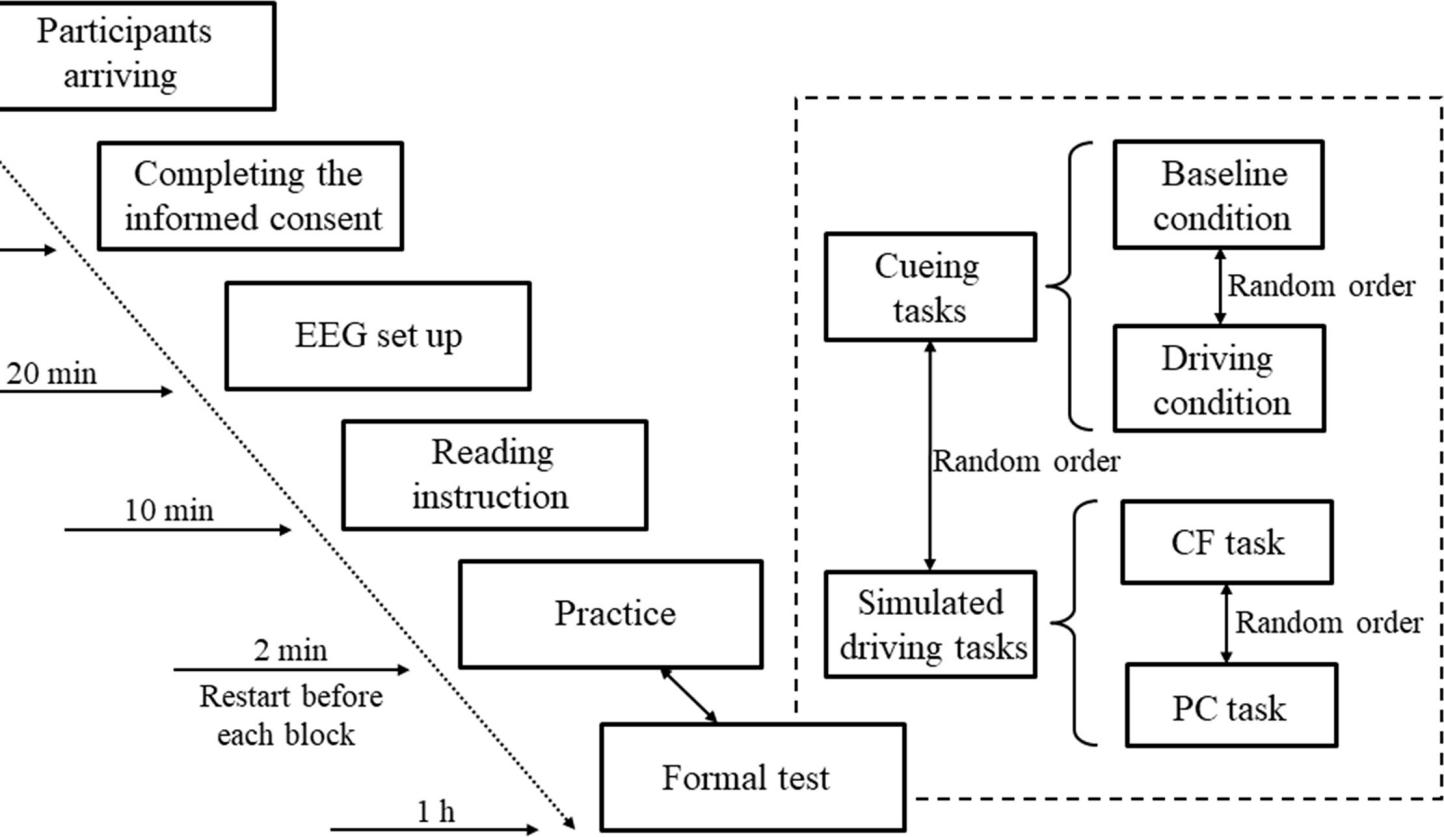
The experimental procedures. The formal testing session consisted of two cueing tasks (cognitive cueing paradigm vs. modified cueing task in simulated driving) and two simulated driving tasks (CF and PC).

The experimental procedure is illustrated in Fig 3. Upon arrival, the participants signed an informed consent form and completed a set of questions regarding their basic demographic information and driving history. Then, with the assistance of two experimenters, participants wore the EEG cap in a dimly lit, electrically shielded room. Following the application of the EEG electrodes, participants read the instructions and started to complete the experimental task, which included 4 blocks. Each block lasted approximately 10 mins. The run order between cueing tasks and simulated driving tasks, as well as the run order within cueing tasks or simulated driving tasks, was counterbalanced across participants. Before each block started, the participants had a short practice (12 trials in cueing tasks and approximately 2 min in simulated driving tasks) to get acquainted with the experimental task and the equipment. In addition, the experimenter checked and ensured that all electrode impedances were less than 5 KΩ. Between each block, there were 5 mins for participants to rest. Only the EEG data recorded in the cueing tasks were used for further analysis.

### Apparatus

**Fig 4:**
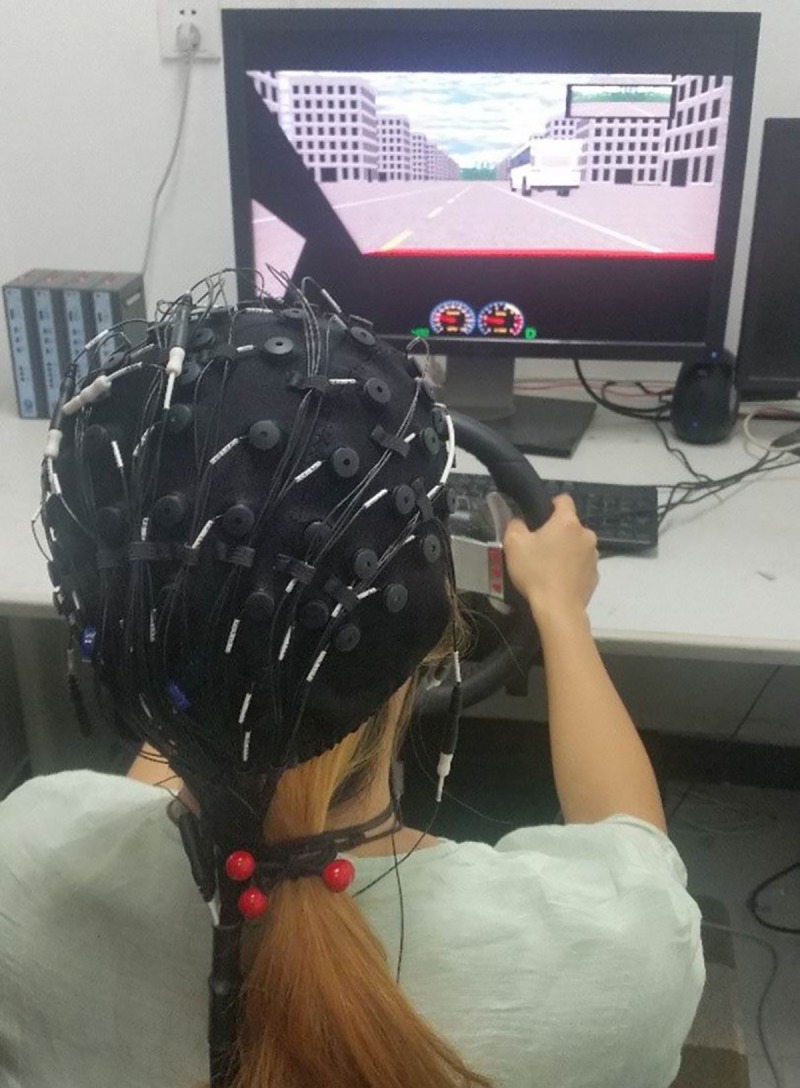
STISIM driving simulator.

The modified cueing task in simulated driving and the two anticipation-related driving tasks were performed using a STISIM^®^ driving simulator (STISIMDRIVE M100K, Systems Technology Inc., Hawthorne, CA; See Fig 4), which was installed on a Dell Workstation (Precision 490, Dual Core Intel Xeon Processor 5130 2 GHz) with a 256 MB PCIe×16 nVidia graphic card, Sound Blaster^®^ X-Fi^™^ system, and Dell A225 Stereo System. A 27-inch LCD screen with 1920×1200 pixel resolution was adopted to display experimental scenarios. The driving simulator also included a force-feedback Logitech Momo^®^ steering wheel, a gas pedal and a brake pedal (Logitech Inc., Fremont, CA).

### EEG acquisition and preprocessing

The EEG signal was acquired using 64 active electrodes attached to an electrode elastic cap (Neuroscan Inc., Charlotte, NC). Electrode positions included the standard International 10–20 system locations and intermediate sites. The four EOG electrodes were placed above and below the left eye and outer canthi of both eyes, and the left mastoid was used as an online reference for all channels. All electrode impedances were less than 5 KΩ. The EEG data were digitized with a sampling rate of 1000 Hz.

The EEG data of the two cueing tasks were processed using Neuroscan 4.5 software. First, EEG data were offline re-referenced to the average of the left and right mastoids and then filtered by means of a FIR filter with a 0.1–30 Hz bandpass. Ocular artifacts were removed from the filtered EEG data using a spatial filter. Afterwards, the onset of the cue was defined as time 0 ms, and the EEG signal was segmented into cue epochs which ranged from -500 to 4000 ms, including a 500 ms pre-cue used for baseline correction. The trials with artifacts larger than +/-100 μV were rejected in any scalp electrodes. Next, EEG epochs were averaged across participants in two conditions. The EEG data of six electrode sites (FC1, FCZ, FC2, C1, CZ, and C2) were used for further analysis. These electrodes were chosen based on the existing literature [37].

### Measurement and data analysis

#### CNV potentials

The CNV was identified as the slow negative wave between the cue and target stimuli, which was divided into two parts for further analysis. According to the literature, the early CNV occurs approximately 1000 ms after the cue stimuli onset and persists for approximately 1000 ms [26, 31]. In present study, the cue stimulus of two cuing tasks was designed to last 3000 ms. Therefore, the early CNV was the average ERP from the 1000 to 2000 ms time window following the cue stimuli, and the late CNV was the average ERP from the 2000 to 3000 ms time window following the cue stimuli. The mean amplitudes of the CNV were calculated by averaging across the two time windows.

For the mean amplitude of the early and late CNV, repeated measures ANOVAs were performed to examine the differences in CNV potentials with cueing condition (cognitive cueing paradigm vs. modified cueing task in simulated driving) and electrode site (FC1, FCZ, FC2, C1, CZ, and C2) as two within-subjects variables. Driving experience (i.e., average driving mileage) which may affect a driver’s expectation of the potential hazards on the road was controlled as a covariate in repeated measures ANOVAs. Significant cueing condition × electrode site interactions were followed up with simple effect analysis to assess the effects that the cueing condition had on the dependent variables for each electrode site.

#### Driving behaviors

The driving behaviors were automatically recorded by the STISIM software at a sampling rate of 10 Hz. In the CF task, the minimum time to collision (min TTC) in all trials was averaged for each participant. The min TTC reflected the time that is available for the driver to avoid a collision with the car ahead and was a critical indicator of the safety outcome of a braking event [35, 38]. In the PC task, only the conditions with crossing pedestrians on both the left and right sides were used, i.e., the minimum speed when the driver was within 130 feet from a pedestrian (min speed of left/right), the speed when a driver passed by a pedestrian (passing speed of left/right), and the distance from the pedestrian when a driver passed by (passing distance of left/right).

To investigate the correlation between CNV and anticipation-related driving behavior, the Pearson correlation coefficients between driving behavior indices and the mean amplitude of early and late CNV at the FC1, FCZ, FC2, C1, CZ, and C2 electrode sites were calculated for the two cueing conditions.

## Results

### Cognitive paradigm of the cueing task

**Fig 5:**
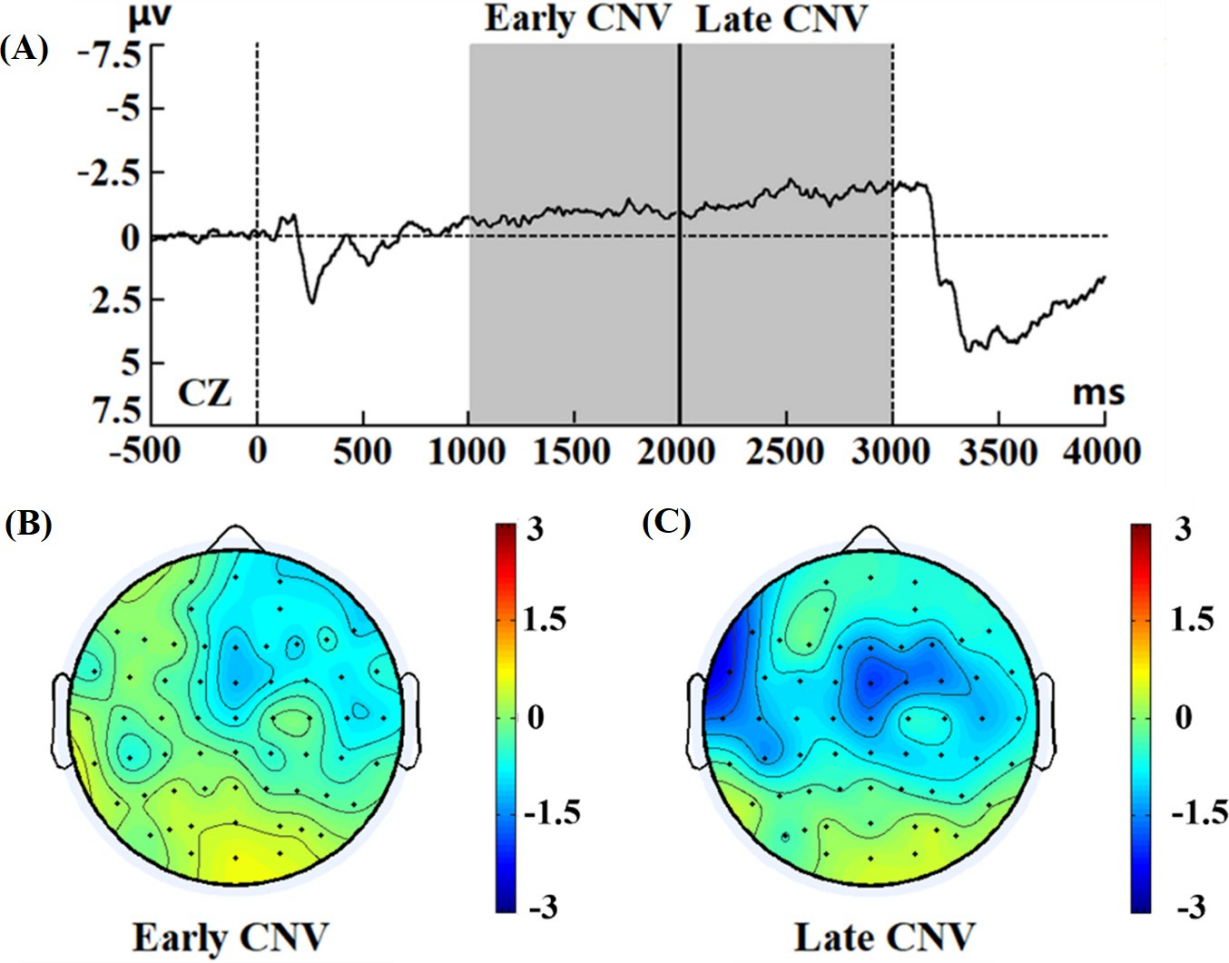
ERP in the baseline condition. (A) Grand average ERP locked to cue stimuli at the CZ electrode. (B) Topographic scalp distribution of CNV mean amplitude between 1000 and 2000 ms, (C) Topographic scalp distribution of CNV mean amplitude between 2000 and 3000 ms.

During the cognitive paradigm of the cueing task, the topographic plots of the average scalp distribution showed that this negativity was maximal at the Cz electrode. Fig 5A shows the EEG grand averages of all participants at the CZ site. Between the presentation interval of the cue and the target stimuli, a negative shift was observed approximately 1000 ms after the presentation of the cue stimuli and continued to increase up to the target stimuli. The average scalp map distribution (1000~2000 ms) (Fig 5B) showed that the early CNV was mainly distributed at the frontal lobe, which was consistent with the early CNV topographic scalp distribution area [19, 30]. The average scalp map distribution (2000~3000 ms) (Fig 5C) showed that the late CNV was spatially localized at the central lobe, which was consistent with the late CNV topographic scalp distribution area [19, 30].

### Modified cueing task in simulated driving

**Fig 6:**
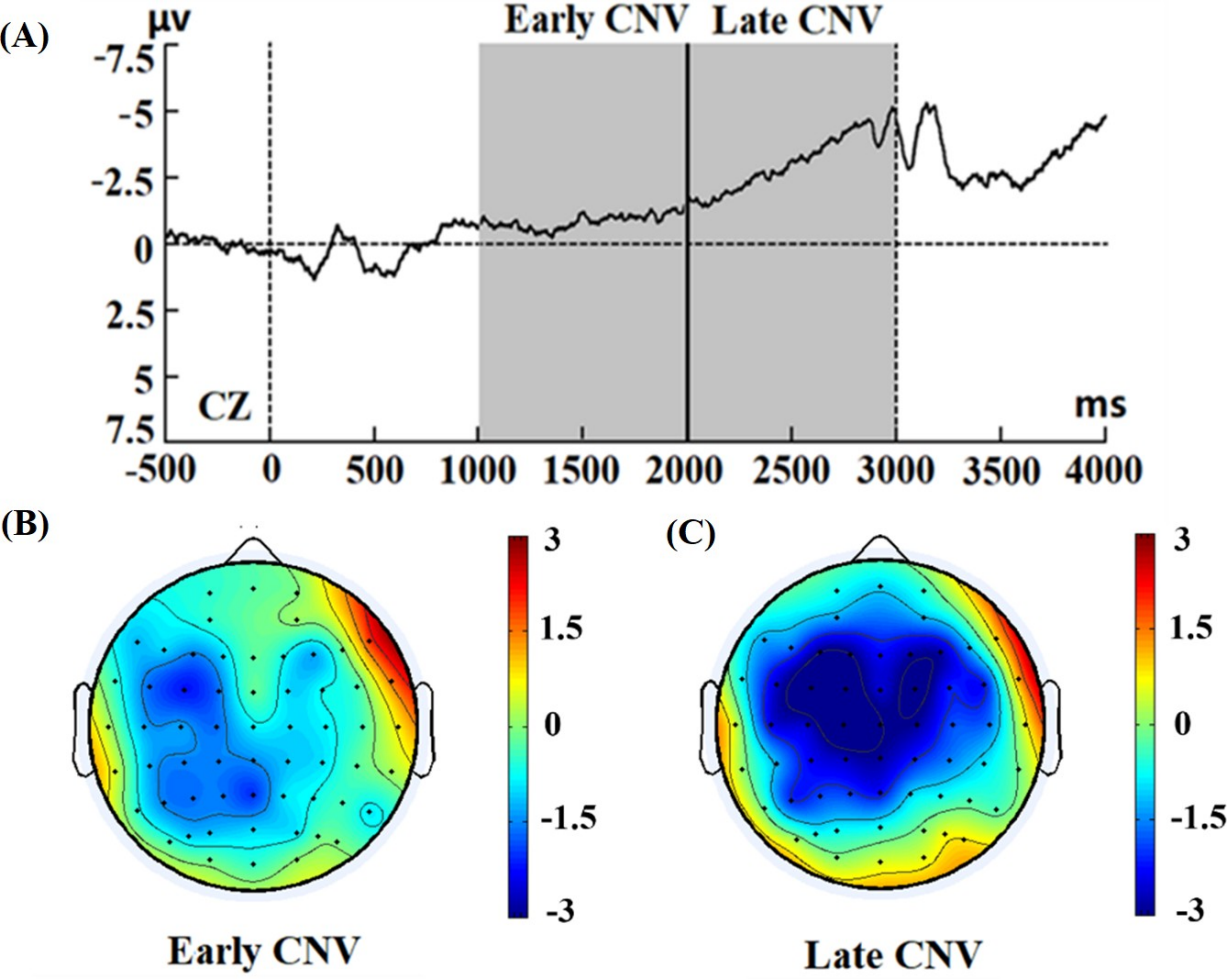
ERP in the driving condition. (A) Grand average ERP locked to cue stimuli at the CZ electrode. (B) Topographic scalp distribution of CNV mean amplitude between 1000 and 2000 ms, (C) Topographic scalp distribution of CNV mean amplitude between 2000 and 3000 ms.

During the modified cueing task in simulated driving, the topographic plots of the average scalp distribution showed that this negativity was maximal at the Cz electrode. The EEG grand averages in the driving condition are shown in Fig 6A. Similar to the baseline condition, there was a negativity induced by the cue stimuli, gradually increasing from approximately 1000 ms after the presentation of the cue stimuli, which is in line with a previous study of movement intention detection [39–41]. Fig 6B presents the topographic scalp distribution of the early CNV (mean amplitude between 1000 and 2000 ms), showing that the early CNV had a broader distribution in driving conditions than in baseline conditions. As shown in Fig 6C, the topographic scalp distribution of the late CNV (mean amplitude between 2000 and 3000 ms) revealed that the late CNV had a similar mean distribution area as the baseline condition, i.e., the central lobe.

### Comparison of CNVs between two cueing tasks

**Fig 7:**
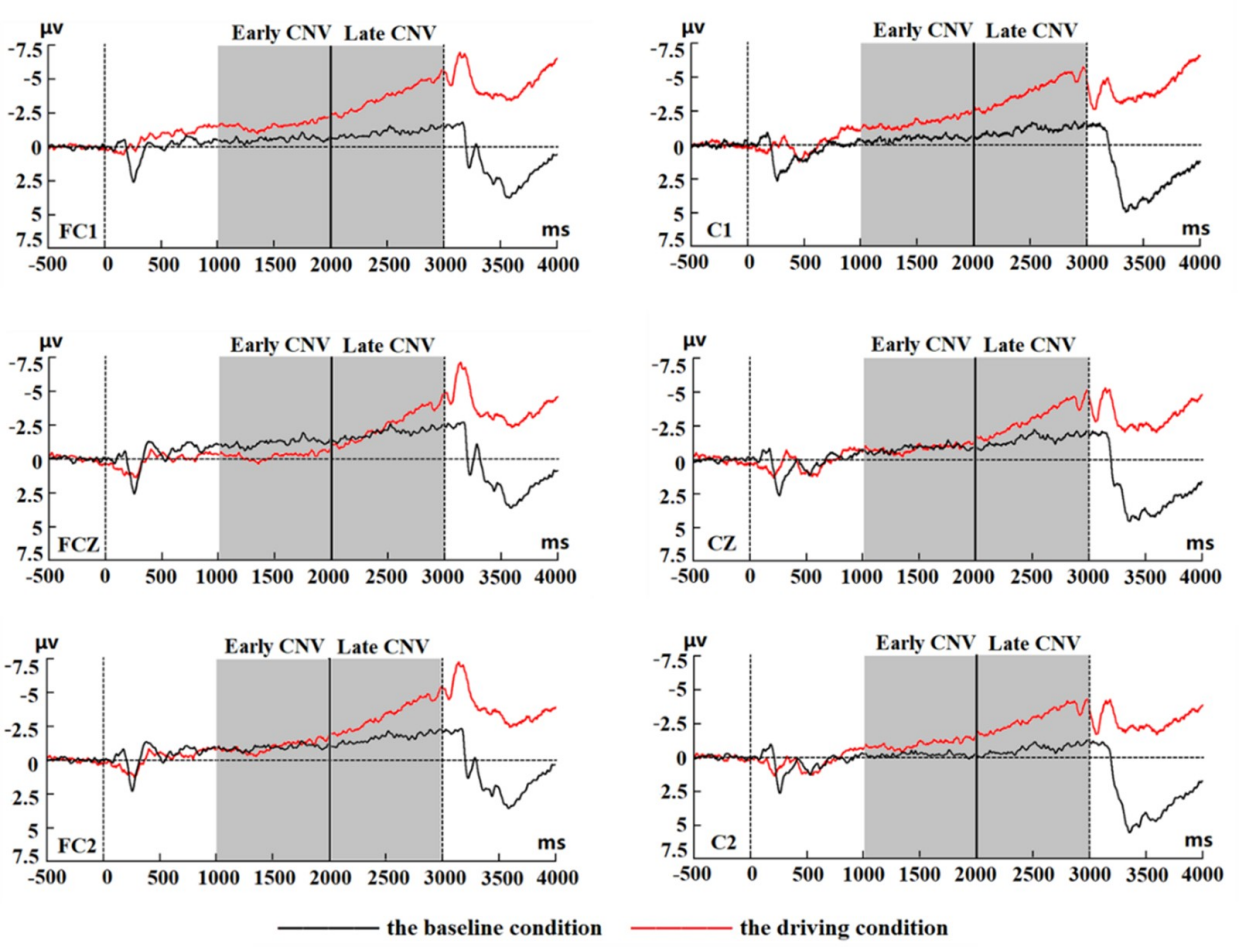
Grand averages ERP locked to cue stimuli at FC1, FCZ, FC2, C1, CZ, and C2 electrodes are shown in black for the baseline condition and in red for the driving condition.

Fig 7 shows the grand-average CNV at the FC1, FCZ, FC2, C1, CZ, and C2 electrodes. The mean amplitude and standard error (SE) of the early and late CNVs are reported in Table 1. The main effect of condition was significant for the early CNV (*F*(1,30) = 4.33, *p* < 0.05, η^2^ = 0.126). The mean amplitude of the early CNV component in the driving condition was more negative than that in the baseline condition. Neither the condition × electrode interaction nor the main effect of electrode was significant.

**Table 1:** The mean amplitude and standard error of early and late CNVs in baseline and driving conditions at FC1, FCZ, FC2, C1, CZ, and C2 electrodes.

Condition	Baseline Condition		Driving Condition	
Electrode site	Early CNV	Late CNV	Early CNV	Late CNV
FC1	-0.57(0.08)	-1.08(0.11)	-1.63(0.06)	-3.76(0.09)
FCZ	-1.21(0.10)	-1.93(0.14)	-0.24(0.08)	-2.56(0.12)
FC2	-0.92(0.09)	-1.64(0.11)	-1.02(0.08)	-3.38(0.12)
C1	-0.46(0.07)	-1.09(0.11)	-1.65(0.13)	-3.96(0.15)
CZ	-0.85(0.11)	-1.54(0.15)	-0.84(0.09)	-3.08(0.12)
C2	-0.18(0.10)	-0.60(0.13)	-1.01(0.12)	-2.90(0.14)

For the late CNV, a significant condition × electrode interaction effect was observed (*F*(5,150) = 2.83, *p* < 0.05, η^2^ = 0.45). Simple effects analyses showed significant differences in four of the six electrodes for the late CNV: FC1 (*F*(1, 30) = 12.23, *p* < 0.01), FC2 (*F*(1, 30) = 5.19, *p* < 0.05), C1 (*F*(1, 30) = 9.11, *p* < 0.01), C2 (*F*(1, 30) = 6.20, *p* < 0.05). The mean amplitudes of the late CNV component in the driving condition were more negative at FC1 (M = -3.76 μv, SD = 2.86 μv), FC2 (M = -3.38 μv, SD = 3.99 μv), C1 (M = -3.96 μv, SD = 4.92 μv), and C2 sites (M = -2.90 μv, SD = 4.50 μv) than those in the baseline condition. In addition, the main effect of electrode was significant for the late CNV (*F* (5, 150) = 3.49, *p* < 0.01, η^2^ = 0.45). There was no significant difference for the late CNV between two conditions.

### Correlation analysis

The Pearson correlation coefficients between the mean amplitude of CNV in driving condition and driving behaviors are shown in Table 2. In the CF task, the min TTC was negatively correlated with the early CNV of driving conditions at FC2, C1, and C2 electrodes and was negatively correlated with late CNV of driving conditions at FC2, C1, and C2 electrodes. In the PC task, the left min speed was positively correlated with the early CNV of driving conditions at the FC1 and C1 electrodes. The right passing distance was negatively correlated with the early CNV of driving conditions at the CZ electrode. No CNV detected in the baseline condition was correlated with driving behaviors.

**Table 2:** The correlation between the mean amplitude of CNV in driving condition and driving behaviors.

CNV	Electrode site	CF Task	PC Task			
		min TTC	left min speed	right min speed	left passing distance	right passing distance
Early CNV	FC1	-0.293	0.373*	0.099	-0.151	-0.229
	FCZ	-0.151	0.096	0.049	-0.135	0.011
	FC2	-0.409*	0.070	0.058	-0.062	0.023
	C1	-0.560**	0.379*	0.256	-0.131	-0.168
	CZ	-0.279	-0.038	-0.181	0.006	0.102
	C2	-0.396*	-0.089	-0.179	0.009	0.061
Late CNV	FC1	-0.195	0.129	0.163	-0.160	-0.205
	FCZ	-0.196	0.106	0.263	-0.183	-0.117
	FC2	-0.379*	0.085	0.137	-0.115	-0.119
	C1	-0.557**	0.321	0.343	-0.141	-0.189
	CZ	-0.302	-0.039	-0.043	-0.027	0.019
	C2	-0.379*	-0.039	0.009	-0.019	-0.033

* Correlation is significant at the 0.05 level.

** Correlation is significant at the 0.01 level.

## Discussion

The present study investigated the existence of CNV in a simulated obscured pedestrian task in the hope of finding a potential way to decrease the risk of obscured pedestrian-motor vehicle crashes. On the one hand, a traditional cognitive paradigm of a cueing task was manipulated as a baseline condition, in which the observed CNV potential was consistent with that in previous studies [42–44]. On the other hand, the modified cueing task (driving condition) was designed in simulated driving scenes, in which CNVs were also observable between the interval of the cue and target stimuli with higher ecological validity.

Due to the relatively long inter-stimulus interval (ISI = 3 s), the early and late components of CNVs were observed in both the baseline and driving conditions. We found larger amplitude of the early CNV in the driving condition than that in the baseline condition. The early CNV, which was affected by the sensory properties of the cue stimuli but not by the target stimuli, mainly reflected the attention to the cue stimuli [18, 45]. In our study, the cue stimuli in both the baseline and the driving condition adopted the visual stimuli. In addition, the cue stimuli were set for the same time duration (3000 ms) and had the same inter-stimulus intervals. Thus, the difference in the amplitude of the early CNV between both conditions may reflect different visual properties of the cue stimuli. The appearance of a park bus at a bus station may activate an individual’s stronger anticipation and action preparation compared to a symbolized person image.

We also found larger negative amplitudes of the late CNV at four of the six electrode sites (FC1, FC2, C1, C2) in the driving condition than those in the baseline condition. The late CNV reflected both anticipation/attention and motor preparation for the target stimuli over frontal and central scalp sites [26, 46, 47]. Schevernels, Krebs [37] speculated that the increased CNV was related to the increased motivation intensity, i.e., additional attention is employed only when it is worth the effort. In the driving condition, if the driver could not avoid the simulated pedestrian successfully, there would be a fierce and lifelike collision frame with a broken front windshield and loud noises. In contrast, in the baseline condition, participants would not receive feedback from their driving behaviors; therefore, no extra motivation would be triggered, and no more processing resources would be used. In addition, Khaliliardali, Chavarriaga [33] conducted a simulated cue brake and cue drive task, finding that the brake trials exhibited a larger negative CNV potential than drive trials in their studies, and they speculated that the difference was caused by the greater movement preparation for brake actions. In the present study, participants in the baseline condition just needed to press a key by using their index finger, whereas in the driving condition, participants were required to avoid a collision and get the car back on the road by steering the wheel appropriately. In other words, the increasing negative CNV elicited in the driving condition might indicate that the obscured pedestrian avoidance scene demanded higher anticipation/attention and motor preparation for the target stimuli than in the baseline condition.

Additionally, correlation analysis showed that some driving behavior indices were significantly correlated with the mean amplitude of CNV elicited in driving conditions. In the CF task, a larger min TTC was related to the more negative CNV waveform of the driving condition. The min TTC reflected the time that is available for the driver to avoid a collision with the car ahead. Previous studies have shown that rear-end collisions were more likely to occur when the lead car braked suddenly and when the drivers did not form adequate anticipation [32, 48]. Indeed, anticipation allowed the driver to control time pressure while driving [49]. In the present study, the results implied that the more drivers could anticipate the braking of the lead car, as reflected by the increasing negative CNV waveform, the greater time margin they would reserve to avoid collision.

The larger left min speed in the PC task were correlated with the less negative CNV elicited in driving conditions, i.e., if the driver could not elicit more negative CNV, their driving behaviors would be more risk. The lessened negative CNV potentials suggested that the driver did not anticipate the movement intention of pedestrians and made inadequate avoidance judgements in avoiding vehicle-to-pedestrian crashes, such as not decelerating or driving too close to the pedestrians. Therefore, the CNV waveform can predict the driving style (safe or risky) of the driver. These correlations between driving behaviors and CNV amplitude suggested that anticipation-related potentials might be a possible predictor of anticipation-related driving behaviors.

To summarize, the obscured pedestrian avoidance task in simulated driving proposed in the present study was successful in eliciting CNV potentials that were more negative than those in the baseline condition due to the stronger motivation and greater motor preparation of drivers in the driving condition. In addition, the mean amplitude of CNV elicited in the obscured pedestrian avoidance task was significantly correlated with several anticipation-related driving behaviors in the two simulated driving tasks, indicating that utilization of the task has the potential to predict the driving safety of drivers. In the future, the task that was employed in this study might be utilized to test and train novice drivers’ crash avoidance abilities, with the elicited CNV amplitude as part of the standard.

The limitation of the current study was that the obscured pedestrian avoidance task was relatively simple and was lack of variety (i.e., the appearance of a parked bus at a bus station was always followed by an obscured pedestrian). The CNV elicited in more flexible and variable conditions (such as unpredictable appearance time of pedestrians or the changeable appearance position of pedestrians) should be investigated in future research. Moreover, future studies may consider to design approaching vehicles in the other lane and compare drivers’ responses to the appearance of the pedestrian in front of the parked bus (e.g., stop at pedestrian crossings or turn toward the left to avoid colliding with the simulated pedestrian).
